# PERCEIVED PHYSICAL FATIGABILITY PREDICTS ALL-CAUSE MORTALITY: THE LONG LIFE FAMILY STUDY

**DOI:** 10.1093/geroni/igz038.3272

**Published:** 2019-11-08

**Authors:** Nancy W Glynn, Theresa Gmelin, Sharon W Renner, Robert M Boudreau, Mary Feitosa, Stacy L Andersen, Kaare Christensen, Anne B Newman

**Affiliations:** 1 University of Pittsburgh, Department of Epidemiology, Pittsburgh, Pennsylvania, United States; 2 University of Pittsburgh Graduate School of Public Health, Pittsburgh, Pennsylvania, United States; 3 University of Pittsburgh, Pittsburgh, Pennsylvania, United States; 4 Washington University School of Medicine, St Louis, Missouri, United States; 5 Boston University School of Medicine, Boston, Massachusetts, United States; 6 Danish Aging Research Center, University of Southern Denmark, Odense C, Denmark

## Abstract

Fatigability, the likelihood of fatigue with lower versus higher levels of exertion, is associated with declines in physical function and disability and related to fitness. Thus, fatigability may be a good predictor of mortality. We examined this relationship in the Long Life Family Study (LLFS), an international family cohort enriched for longevity and their spousal controls. We measured perceived physical fatigability at Visit 2 (2014-2017) using the Pittsburgh Fatigability Scale (PFS, 0-50 with higher score=greater fatigability). We identified deaths by family members notifying field centers, reporting during annual phone follow-up, or finding an obituary when unable to reach. Otherwise, we censored participants at most recent contact date when confirmed alive. Covariates included age, sex, and self-reported physical activity using the Framingham Physical Activity Index. We adjusted all analyses for field center and family structure. Participants alive ≥60 years (range 60-108, mean 73.6±10.5) and completed the PFS (N=2,326) at Visit 2 were predominantly white (99.5%) and female (55.1%). Post-Visit 2, 195 (8.4%) died during mean 2.5±1.0 years of follow-up. Age-adjusted PFS score was 7.7 points greater (p<.0001) for those who died (19.8) compared to alive (12.1). Using Cox Proportional-Hazard modeling, each 5-point greater PFS score was associated with 31% (HR: 1.31, 95% CI 1.18,1.43) higher all-cause mortality rate adjusted for covariates listed above. Further adjustment for comorbidities did not attenuate association. PFS’s perceived physical fatigability score may be a useful self-report clinical tool to predict higher risk of mortality among older adults when objective measures of fitness and function are unavailable.

